# Supplementary figures and images for: Transcriptional Profiling Defines Unique Subtypes of Transit Amplifying Neural Progenitors Within the Neonatal Mouse Subventricular Zone
Source: Biomolecules. 2025 Oct 11;15(10):1438. doi: 10.3390/biom15101438 (PMC12563026; doi:10.3390/biom15101438)

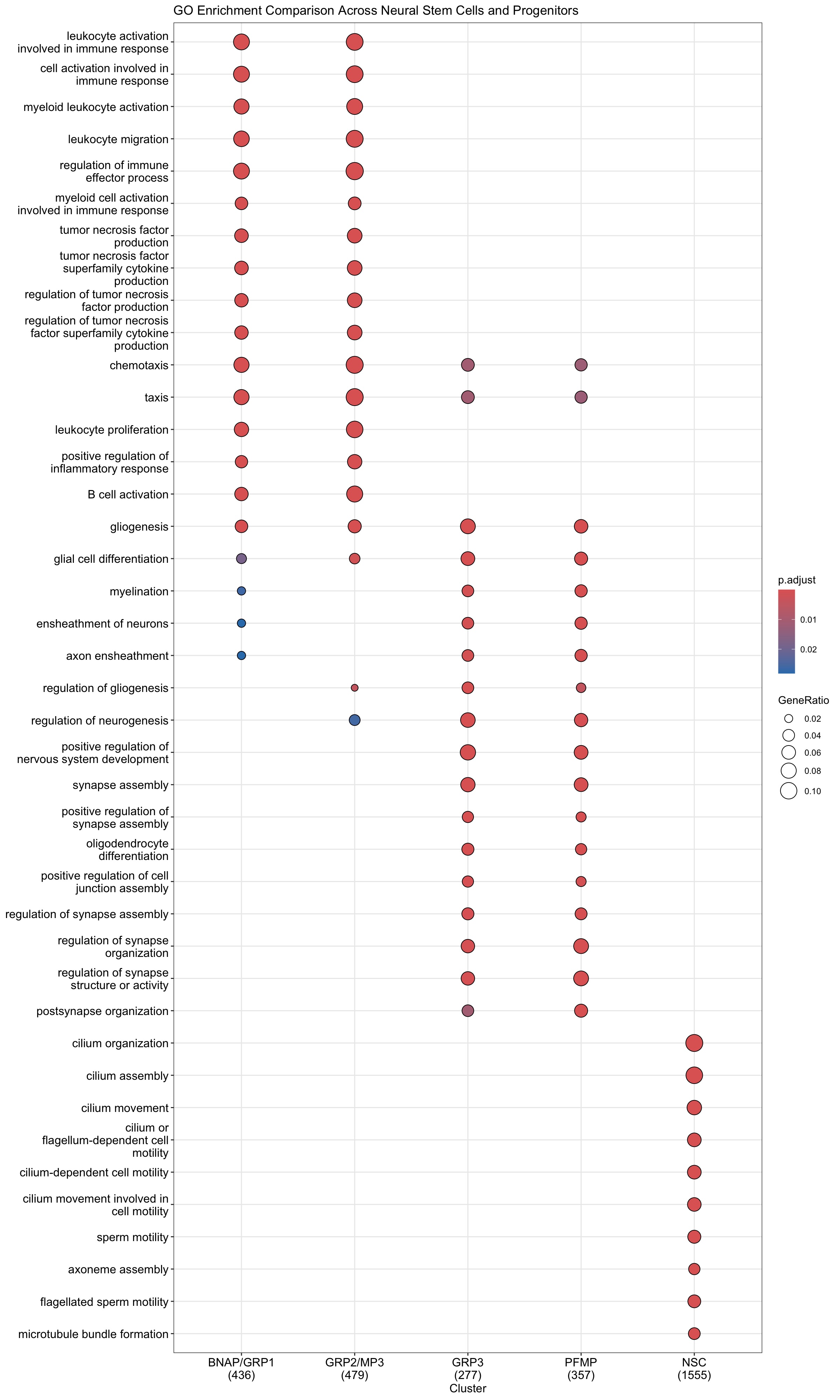

Supplement: Supplementary file 1 [file biomolecules-15-01438-s001.zip › biomolecules-3874024-supplementary/ZaritskyKumari_SupFigure1.jpg]
